# Prevalence of Occult Hepatitis B Virus in Plasma and Peripheral Blood Mononuclear Cell Compartments of Patients With Chronic Hepatitis C Infection in Tehran-Iran

**DOI:** 10.5812/hepatmon.10134

**Published:** 2013-05-29

**Authors:** Zeinab Vakili Ghartavol, Seyed Moayed Alavian, Safieh Amini, Rouhollah Vahabpour, Golnaz Bahramali, Ehsan Mostafavi, Mohammad Reza Aghasadeghi

**Affiliations:** 1 Department of Basic Sciences, Science and Research Branch, Islamic Azad University, Tehran, IR Iran; 2 Baqiyatallah Research Center for Gastroenterology and Liver Diseases, Baqiyatallah University of Medical Sciences and Tehran Hepatitis Center, Tehran, IR Iran; 3 Department of Hepatitis and AIDS, Pasteur Institute of Iran, Tehran, IR Iran; 4 Department of Epidemiology, Pasteur Institute of Iran, Tehran, IR Iran; 5 Department of Virology, Tehran University of Medical Sciences, Tehran, IR Iran

**Keywords:** Hepatitis B, Hepatitis C Virus, Peripheral Blood Mononuclear Cell

## Abstract

**Background:**

Occult hepatitis B virus (HBV) infection (OBI) is frequently reported in patients with chronic hepatitis C virus (HCV) infection. An association between OBI and more liver damage, cirrhosis, hepatocellular carcinoma, and reduced response to interferon therapy in patients with HCV infection is suggested.

**Objectives:**

The aim of this study was to determine the prevalence of occult HBV, and evaluate its clinical influence on patients with chronic HCV.

**Patients and Methods:**

A cohort study including50 patients with positive results for HCV, and negative results for HBsAg tests was performed. The patients were divided into two groups: one group had positive results for both HCV and occult HBV tests (n = 18), and the other had positive results for HCV, but negative findings for occult HBV (n = 32). All were treated with PEG-IFN alpha-2a and Ribavirin. Presence of HCV RNA was followed in these patients.

**Results:**

HBV-DNA was detected using nested-PCR in 20% of plasma and 32.6% of peripheral blood mononuclear cell (PBMC) compartments. No significant differences were observed between patients with and without occult HBV for sex, age, duration of HCV infection, histological markers, presence of anti-HBc, HCV viral load, and HCV genotype. The response rate was significantly higher in patients with positive results for HBV-DNA test compared to those with negative findings (100% vs. 71.9 %, P < 0.05).

**Conclusions:**

In conclusion, occult HBV was found in 36% of patients with negative results for HBsAg, but positive results for HCV. Detection of HBV-DNA in both PBMCs and plasma together in comparison with plasma alone provided more true identification of OBI.The SVR rate was significantly higher in coinfected patients than mono-infected ones.

## 1. Background

Hepatitis B virus (HBV) and hepatitis C virus (HCV) infections are the cause of liver diseases in the significant proportions worldwide (1). Since both viruses have similar transmission routes, prevalence of current or previous HBV coinfection among patients with HCV infection is common (2). However, the persistent presence of hepatitis B surface antigen (HBsAg) in blood is demonstrated as the overt infection by HBV. HBV infection with negative results for HBsAg test, or occult HBV infection (OBI) is a recently clinical identified entity, which is also frequently recognized in patients with chronic HCV infection (3). Recent studies showed variable prevalence of OBI among patients with HCV from 0% to 52 % (4). OBI mainly was found in association with the long-lasting intrahepatic and extrahepatic (peripheral blood mononuclear cells) (PBMCs) persistence of hepatitis B virus-DNA (5, 6) and in some instances, in the sera of cases with negative results for hepatitis B surface antigen (HBsAg) (7). It also correlated with a strong suppression of viral replication and gene expression (8) in individuals with or without serological markers of previous HBV infection (anti-HBc and anti-HBs) (5). Because of very low viral levels in occult hepatitis B, using very sensitive molecular tests such as nested-PCR, real-time PCR, and transcription based mediated amplification (TMA) (7-9) is very important for its complete identification that could provide a lower limit of detection of less than 10 IU/ML for HBV DNA (10). The true clinical impacts of occult HBV in patients with chronic HCV are greatly unknown. Some studies suggested that presence of occult HBV could be associated with more severe liver damage (3, 11, 12), cirrhosis (13) and increased rate of hepatocellular carcinoma (14). OBI has also been regarded as a cause of interferon therapy failure in some (3, 15, 16), but not all studies (17, 18). The role of OBI also remains unclear in association with liver enzymes flare.

## 2. Objectives

The aim of this study was to detect the presence of occult hepatitis B in the plasma and the PBMCs of a cohort of 50 patients with chronic HCV infection using nested PCR, and also to assess the clinical consequences of occult HBV in these patients.

## 3. Patients and Methods

### 3.1. Patients and Samples

Using convenience sampling, a group of 50 patients with HCV infection attending the Tehran Hepatitis Center (Iran), to start their HCV infection therapy during March 2010 to April 2011 were selected. The patients included 34 males and 16 females with the mean ± SD ages of 31.50 ± 8.06 and 22.62 ± 5.20 years, respectively. Inclusion criteria included positivity for anti-HCV, serum HCV-RNA detection, and undetectable HBsAg in serum. None of the study patients were infected by human immunodeficiency virus (HIV). The first samples were taken before treatment, and the second ones were drawn 12 weeks after therapy. Based on the nested-PCR results, the patients were divided into two groups: one group had positive results for both HCV and occult HBV tests (n=18), and the other had positive results for HCV and negative results for occult HBV (n=32). The study patients had two types of HCV genotype 1a or 3a, and all were treated with PEG-IFN alpha-2a (Pegasys, 180 microgram per week) and Ribavirin (800-1200 mg, daily, according to weight). Patients with HCV were also stratified according to the reduction of HCV RNA levels in serum: rapid virologic response (RVR) showing undetectable serum HCV-RNA at 4 weeks after starting therapy, early virologic response (EVR) was defined as the disappearance or reduction in serum HCV-RNA levels by at least 2 log10 at 12 weeks after starting therapy, and nonearly virologic response (non-EVR), as a lack of reduction in HCV-RNA serum by more than 2 log10 at 12 weeks in comparison with the pretreatment levels (19). We analyzed virological response rate during therapy (RVR, EVR and non-EVR) regarding the presence of HBV-DNA. We collected second serum and PBMC samples from 22 available patients to find out the presence of HBV DNA in relation to HCV RNA levels. Sixteen patients achieved RVR or EVR, and had negative findings for HCV RNA. The remaining 6 patients were non-EVR with lower HCV RNA, and negative results for HBV DNA before therapy.

### 3.2. Routine Analysis

HBV serum markers (HBsAg and anti-HBc,) were determined by ELISA using commercially available kits (Dia Pro. Diagnostic Italy). HCV-RNA in serum and PBMC before and during therapy, were quantified with the COBAS Taq Man HCV (Roche Diagnostics).

### 3.3. Histological Diagnosis

The liver biopsies were available from 44 patients before antiviral therapy to evaluate the degree of histological lesion. A numerical score was calculated for each liver biopsy specimen, both for grading necroinflammatory activity (Histological Activity Index, HAI) and the degree of fibrosis (stage). The grade and stage was determined according to Ishak Modified scoring system (20). All liver biopsy specimens were fixed and paraffin embedded for histological analysis if they were sufficient for evaluation of liver staging and grading, but available samples for diagnosis of HBcAg using immunohistochemical test were limited only to 13 liver biopsies.

### 3.4. Isolation of Plasma and PBMCs

Before and during the antiviral therapy, a volume of 4-5 ml blood sample from each patient was drawn and transferred to EDTA tube. Plasma was isolated and saved at -20 °C until used. PBMCs were isolated from blood by gradient centrifugation using Lympholyte®-H kit (Cedarlane Laboratories, Canada). After isolation, the cells were washed twice with phosphate-buffered saline (PBS, pH 7.2) and suspended in 200-400 µL RNA-later (RNA-later Stabilization Reagent QIAGEN kit), and stored at -20 ^°^C to evaluate HBV-DNA.

### 3.5. HBV-DNA Detection

For each patient, plasma and PBMCs were explored for the presence of HBV-DNA. Viral DNA was extracted from 200 µl of plasma and PBMCs in RNA-later using High Pure Viral Nucleic Acid Kit (Roche Diagnostics).Nested-PCR was used to detect HBV-DNA in plasma and PBMCs, two pairs of oligonucleotide primers provided in accordance with the highly conserved S/pol region of HBV genome were used for detection of S region (21).

The sequences of the primers used in Nested PCR included:

Outer-YMDD-F: 5´-GGTTATCGCTGGATGTGTCTGC-3´ :365-386

Outer-YMDD-R: 5´-CCACAATACGTTGACAGACTTTCC-3´ : 980-1004

Inner-YMDD-F: 5´-CTCTTCATCCTGCTGCTATGCCTC-3´ : 404-425

Inner-YMDD-R: 5´-TGGTAACAGCGCTAAAAAGGGACTC-3´ : 781-805

The PCRs were performed in a 25 µl reaction volume containing 5 µl of DNA sample for the first round PCR, and 2 µl PCR product for the second round PCR, and 10 pmol of each primer in each round PCR that performed with master kits (iNt Ron, KOREA). The amplification protocol for the two successive rounds of PCR included: first round with 30 cycles, each cycle contained denaturation at 940C for 40 sec, annealing at 650C for 40 sec, and extension at 720C for 40 sec, and the second round with 35 cycles, each cycle contained denaturation at 940C for 25 sec, annealing at 660C for 25 sec, and extension at 720C for 25 sec with the final extension step at 720C for 3 min. The amplified products were visualized in 1% agarose gel stained with Ethidium bromide. We used HBV-DNA positive controls (106copies/mL) and negative control containing serum samples and water at each PCR. The cutoff of HBV DNA detection was 5 IU/ml. All samples were tested for HBV DNA detection using nested-PCR assay in duplicate. We regarded the cases that showed positive results in duplicate.

### 3.6. Immunohistochemical Examination

Paraffin-embedded liver specimen was cut into 3-4µ sections and mounted on microscope slides for analysis. Immunohistochemical (IHC) was performed in two steps. First step, included deparaffinizing at 600C and dehydrating in xylene, alcohol series (1000, 960,700, 500) for 3 minutes at room temperature, and was washed in PBS twice for 4 minutes. Second step included adding antibodies, according to protocol (EnVision ^TM^ + Dual Link System-HRP Dako) kits administrated. Briefly, sections were treated with blocking reagent for inhibition of peroxidase activity. None-specific binding was blocked by normal rabbit serum for 30 minutes. Then polyclonal anti-HbcAb was applied as primary antibody and incubated in a moist chamber for two hours. Then visualization process was performed according to the manufacture (EnVision ^TM^ + Dual Link System-HRP Dako) kits. Negative and positive controls were used simultaneously to ensure reliability and specificity.

### 3.7. Statistical Analysis

Statistical analysis was conducted with SPSS 16 software. The results were expressed as percentages, means ± SD or median (max-min). Groups were compared using the chi-square test for categorical variables and student’s t-test, analysis of variance (ANOVA) and multivariate analysis for quantitative variables. All comparisons were two-tailed, and p-values less than 0.05 were considered to be statistically significant Mann-Whitney U test was used for comparing the continuous variables that did not have normal distribution.

## 4. Results

HBV-DNA was detected in 18 (36%) of 50 patients with negative results for HBsAg, and positive results for HCV. Of these 18 patients, 7 (38.9%) had positive findings in both plasma and PBMCs, 8 (44.4%) in only PBMCs, and 3 (16.7%) in plasma samples only (data not shown). All these samples were collected before treatment (first samples). There were no statistical difference in gender, age, duration of HCV infection, HCV viral load and HCV genotypes in patients with or without occult HBV. Also, no significant difference for the presence of anti-HBc was observed in patients with positive or negative results for HBV-DNA (Table 1).

**Table 1.** Demographic data of patients with chronic hepatitis C with and without occult HBV

Data	Occult HBV (+) (n = 18)	Occult HBV (-) (n = 32)	P value
**Age, Mean ± SD, y**	29.88 ± 8.30	27.96 ± 8.41	0.44
**Sex, Male/Female**	13/5	21/11	0.75
**ALT(pre)^a^, Mean ± SD, IU/mL**	47.23 ± 32.17	70.77 ± 43.11	0.05
**AST(pre)^a^, Mean ± SD, IU/mL**	41.29 ± 25.28	84.41 ± 74.03	0.005^c^
**ALKP(pre)^a^, Mean ± SD, IU/mL**	214.50 ± 88.62	316.45 ± 138.49	0.004^c^
**ALT at 12 Weeks^b^,Mean ± SD, IU/mL**	32.37 ± 25.44	49.13 ± 33.14	0.08
**AST at 12 Weeks^b^,Mean ± SD, IU/mL**	36.44 ± 27.10	49.67 ± 35.22	0.19
**ALKP at 12 Weeks^b^,Mean ± SD, IU/mL**	292.44 ± 188.71	303.64 ± 125.24	0.81
**Duration of HCV Infection, Mean ± SD,y**	5.60 ± 3.72	6.32 ± 4.83	0.59
**Anti-HBc(+)/Total number (%)**	3/14 (21.4)	7/27 (25.9)	0.75
**HCV genotype (%)**			
1^b^	12 (70.6)	20 (62.5)	0.57
3^b^	5(29.4)	12 (37.5)	
**HCV Viral Load,Mean ± SD, IU/mL**	8.79×10^5^± 8.74×10^5^	9.26×10^5^± 10.10×10^5^	0.87
**Histological Activity**			
Stage, Median (Min-Max)	3.50(0-6)	4.40(0-6)	0.54
Grade, Median (Min-Max)	7.15(0-14)	7.80(0-14)	0.43

^a^Abbreviations: ALT, alanine transaminase; AST, aspartate aminotransferase; ALKP, Alkaline Phosphatase; Pre, Pretreatment

^b^12 weeks after starting therapies

^c^Statistically significant

Of 50 patients with chronic HCV treated with peg-interferon and ribavirin combination, 21 became undetectable for HCV-RNA at 4 weeks, and they achieved an RVR. Serum HCV-RNA also remained undetectable at 12 weeks after starting therapy in these patients. Serum HCV-RNA became undetectable in further 16 patients at 12 weeks after starting treatment, and they achieved a complete EVR. In 4 patients, the serum HCV-RNA levels did not become undetectable but reduced by ≥ 2 log10 and these patients achieved a partial EVR. In these patients, HCV- RNA levels became undetectable between 12 and 24 weeks. In nine patients, the reduction in serum HCV RNA levels was < 2 log10, and they had a non-EVR, and also remained detectable for HCV-RNA levels at 24 weeks after starting therapy, that according to the American guidelines, detectable HCV-RNA serum at 24 weeks after beginning therapy was described as a nonresponse (i.e. null response or partial nonresponse) (22).

Moreover, we did not observe significant differences in histological grading and staging when comparing patients with detectable to those undetectable for HBV-DNA (Table 1). The mean ALT, AST and ALKP levels before starting therapy were significantly higher in patients with negative results for occult HBV than those with positive findings (Table 1). Considering virological responses (RVR, EVR and non-EVR) between before and 12 weeks after starting therapy, a poor decrease on AST levels (P = 0.09) was detected in cases with OBI (Median: 13; Range: 147) and without OBI (Median: 9.5; Range: 202). There was no difference in ALT and ALKP levels between the two groups. The rate of virological response during therapy (RVR, EVR and non-EVR) in relation to presence of occult HBV was available for 41 of 50 (82%) patients with HCV infection. Of these 21 achieved an RVR and 20 an EVR; while, serum HCV RNA remained undetectable at 24 weeks after starting therapy in all these patients. The response rate was significantly higher in patients with positive results for HBV-DNA compared to those with negative results (100% vs. 71.9 %, P < 0.05) (Table 2). Nine (28.1%) of cases without HBV-DNA showed non-EVR during therapy and HCV RNA remained detectable at 24 weeks after starting treatment in these patients (Table 2). Also, the patients who showed a virologic response during therapy (RVR or EVR) had significantly lower duration of HCV infection than patients who showed non early virologic response (non-EVR) (P = 0.04) (Table 2).

**Table 2.** Impact of Virological and Epidemiological Features on Virologic Response During Therapy

Data	RVR (n = 21)	EVR (n = 20)	Non-EVR (n = 9)	P value
**HCV Viral Load^a^, Mean ± SD, IU/mL**	5.00×10^5^± 8.36×10^5^	1.27×10^6^± 1.09×10^6^	1.09×10^6^± 0.48×10^6^	0.02^b^
**Genotype (%)**				0.17
1^a^	10 (31.2)	15 (46.9)	7 (21.9)	
3^a^	10 (58.8)	5 (29.4)	2 (11.8)	
**Duration of HCV Infection^c^, Mean ± SD, y**	4.90 ± 4.34	5.91 ± 3.74	9.37 ± 4.95	0.04^b^
**HBV-DNA**				0.04^b^
HBV-DNA positive (%)^d^	9 (50)	9 (50)	0 (0.0)	
HBV-DNA negative (%)^e^	12 (37.5)	11(34.4)	9 (28.1)	

^a^HCV viral load are baseline values

^b^Statistically significant

^c^Defined between the first times of diagnosis of HCV infection and beginning therapy in our study

^d^Total response rate (RVR + EVR) in cases with positive results for HBV-DNA was 100 %

^e^Total response rate in (RVR + EVR) cases with negative results for HBV-DNA was 71.5 %

Likewise, HCV viral load in patients with RVR was significantly lower than patients with non-EVR, irrespective of the HBV-DNA status (P = 0.02). No statistically significant difference was observed in the rate of virological response during therapy regarding HCV genotypes (Table 2). Second plasma samples from 6 patients with EVR, stayed HBV-DNA positive and 7 (4 with EVR and 3 with non-EVR) having no HBV-DNA in their first plasma samples, showed positive results in their second samples. HBV-DNA remained negative in 8 cases (5 with EVR and 3 with non EVR) similar to their first plasma samples. Also, HBV DNA was detected in PBMCs of 13 patients similar to their first samples.

Immunohistochemical study in 13 liver biopsies samples showed HBcAg in 3 of them, two of these cases had positive results for HBV-DNA in plasma and/or PBMCs compartments, and one patient had negative findings in both samples.

## 5. Discussion

In this study, the prevalence of occult HBV in patients with chronic HCV was 20% (10 of 50) in plasma and 32.6% (15 of 46) in PBMCs samples. This observation showed that exploring only plasma samples is not sufficient to identify occult HBV infection, and the more reliable information was obtained by examining both the plasma and PBMCs compartments, especially when the liver specimens were not available for detection of HBV-DNA. Therefore, PBMC may be an alternative source to liver biopsy for detection of an occult HBV infection. This result also was comparable to Sagnelli et al study who showed that nearly a half of HCV patients with detectable HBV-DNA in PBMCs had negative results in plasma (23 vs. 12 cases) (23). Presence of occult HBV has been reported in association with the presence of markers of HBV exposure (anti-HBc ± anti-HBs). But we found no correlation between the presence of anti-HBc and prevalence of HBV-DNA in HBsAg-negative and HCV positive patients, this was compatible with some studies (3, 24). However, other studies showed higher prevalence of occult HBV among individuals seropositive for anti-HBc and/or anti-HBs, especially those with the presence of anti-HBc alone, compared with patients who had not positive results for anti-HBc and anti-HBs (1, 17, 23). The clinical significance of occult HBV is still not understood and the overall trend in the literature shows inconsistent results. To evaluate the clinical influence of occult HBV in patients with chronic HCV, we compared histological parameters with the presence of HBV-DNA. We found no statistically significant difference respecting inflammatory activity (grading) or hepatic fibrosis (staging) in studying patients with chronic hepatitis C with and without occult HBV infection as were reported by some other researchers (17, 18). Nonetheless, in accordance with other studies, the liver disease in patients with chronic HCV with occult HBV can advance at a higher rate than those with HCV infection alone (3, 11, 12, 25). However, different criteria that regarded for selecting patients may explain this discrepancy. For example, our study population consisted younger individuals (29.88 ± 8.30) than Mrani et al., study population (45 ± 12) (3). The younger age of our patients causing comparatively shorter duration of HCV infection in our cases may preclude the complete identification of significant association between OBI and histological progression. The presence of HBV-DNA and consequently high liver enzymes levels in patients with chronic HCV is also controversial. Our findings demonstrated the elevation of liver enzymes levels in HCV patients with negative results for HBV-DNA. While the HCV patients who had positive findings for HBV-DNA, showed normal or slightly increased liver enzymes levels. These findings were comparable with Chen et al., study who showed that HCV patients with occult HBV had lower ALT levels than those with HCV infection alone (2). Likewise, significant reduction of liver enzymes levels at 12 weeks, due to HCV antiviral therapy in our patients with positive results for HCV and negative results for HBV-DNA showed that ALT and AST levels correlate with serum HCV-RNA levels. These observations could be explainable with the viral interference between HCV and HBV. As cotransfection experiments with HCV and HBV showed that the secretion of HCV RNA levels can be decreased by HBV DNA (26). Therefore, our results indicated that presence of HBV-DNA could cause an inhibitory influence on elevation of liver enzymes, and provides additional support for the opinion that HBV-DNA has inhibitory interference on HCV activity. Also, the result of normal liver enzymes in our patients with OBI were comparable to many studies that failed to demonstrate the association between OBI and elevation of liver enzymes in patients with HCV (17, 24, 27, 28). However, other studies reported a direct correlation between the presence of OBI and flare liver enzymes (29, 30). In the present study, seven patients (4 patients who achieved an EVR and 3 a non EVR) with negative results for HBV-DNA in first plasma, showed appearance of HBV-DNA in second plasma following clearance or reduction of serum HCV-RNA levels due to HCV antiviral therapy. This observation suggests diminishing inhibitory effect of HCV-RNA levels on HBV-DNA, since occult HBV could replicate and be detectable in plasma. Indeed, the concept of viral interference is one of the reasons that may affect HBV replication and gene expression. HBV replication in chronic HCV infection with concurrent occult HBV infection could be suppressed by HCV core protein (10, 24, 31-33) which may be reversible and occult infection may reactivates, developing classical hepatitis B (4). Therefore, it is possible that HBV flares up when the HCV virus is treated (34). However, the fluctuation of HBV replication could not be excluded (4). Nevertheless, serial follow-up PCR examinations are needed to exclude dual HBV and HCV infection. We found that all patients with positive results for occult HBV showed a higher virological response rate during therapy compared to those with negative findings, and all patients who showed non early virological response had negative results for occult HBV before treatment. These results indicated that patients with occult HBV had more decrease in HCV-RNA levels than negative ones. This is comparable with the study of Kazemi Shirazi et al. who reported that patients with chronic HCV and HBV-DNA had (P = 0.009) negative results for HCV-RNA more common than those without HBV-DNA (35). So, this result suggests that HBV genome could also possibly suppress HCV replication. Overall, these findings may hypothesize mutual interference between HBV and HCV viruses in our study patients. In addition, the patients who achieved a virologic response during therapy (an RVR and EVR) had lower duration of HCV infection and HCV viral load than nonearly virologic responders, regardless of HBV-DNA status. Thereupon, these data raise some doubt as to whether disappearance of serum HCV-RNA levels is affected by an interaction between two viruses, or whether other factors also have interfered. Finally, examination of 13 HBV-DNA positive second plasma samples showed that despite of HCV antiviral therapy and a virologic response rate during therapy, HBV-DNA could be persistent and even with decrease or disappearance of HCV genome may raise. This showed that HBV-DNA was not sensitive to PEG-IFN and Ribavirin functions as reported in the study of Khattab et al. (1). Also, the presence and persistence of HBV-DNA in PBMCs may infect liver again, and cause the relapse of hepatitis (36). In conclusion, OBI was found in a considerable number of plasma and PBMCs of Iranian patients with chronic hepatitis C infection with undetectable HBsAg, irrespective of the anti-HBc status. Detection of HBV-DNA in both PBMCs and plasma together in comparison with plasma alone provided more true identification of OBI. Furthermore, the presence of HBV-DNA was found in association with normal liver enzymes levels and more decrease in HCV- RNA loads in comparison with patients with negative results for HBV-DNA. This warrants further studies with more patients, considering different host and viral factors simultaneously, are needed to confirm these data.

## References

[1] Khattab E, Chemin I, Vuillermoz I, Vieux C, Mrani S, Guillaud O (2005). Analysis of HCV co-infection with occult hepatitis B virus in patients undergoing IFN therapy.. J Clin Virol..

[2] Chen LW, Chien RN, Yen CL, Chang JJ, Liu CJ, Lin CL (2010). Therapeutic effects of pegylated interferon plus ribavirin in chronic hepatitis C patients with occult hepatitis B virus dual infection.. J Gastroenterol Hepatol..

[3] Mrani S, Chemin I, Menouar K, Guillaud O, Pradat P, Borghi G (2007). Occult HBV infection may represent a major risk factor of non-response to antiviral therapy of chronic hepatitis C.. J Med Virol..

[4] Levast M, Larrat S, Thelu MA, Nicod S, Plages A, Cheveau A (2010). Prevalence and impact of occult hepatitis B infection in chronic hepatitis C patients treated with pegylated interferon and ribavirin.. J Med Virol..

[5] Fernandez-Rodriguez CM, Gutierrez ML, Lledo JL, Casas ML (2011). Influence of occult hepatitis B virus infection in chronic hepatitis C outcomes.. World J Gastroenterol..

[6] Carreno V, Bartolome J, Castillo I, Quiroga JA (2008). Occult hepatitis B virus and hepatitis C virus infections.. Rev Med Virol..

[7] Ocana S, Casas ML, Buhigas I, Lledo JL (2011). Diagnostic strategy for occult hepatitis B virus infection.. World J Gastroenterol..

[8] Raimondo G, Pollicino T, Cacciola I, Squadrito G (2007). Occult hepatitis B virus infection.. J Hepatol..

[9] Fang Y, Shang QL, Liu JY, Li D, Xu WZ, Teng X (2009). Prevalence of occult hepatitis B virus infection among hepatopathy patients and healthy people in China.. J Infect..

[10] Hollinger FB, Sood G (2010). Occult hepatitis B virus infection: a covert operation.. J Viral Hepat..

[11] Sagnelli E, Coppola N, Scolastico C, Mogavero AR, Filippini P, Piccinino F (2001). HCV genotype and "silent" HBV coinfection: two main risk factors for a more severe liver disease.. J Med Virol..

[12] Tamori A, Nishiguchi S, Kubo S, Enomoto M, Koh N, Takeda T (2003). Sequencing of human-viral DNA junctions in hepatocellular carcinoma from patients with HCV and occult HBV infection.. J Med Virol..

[13] Pollicino T, Squadrito G, Cerenzia G, Cacciola I, Raffa G, Craxi A (2004). Hepatitis B virus maintains its pro-oncogenic properties in the case of occult HBV infection.. Gastroenterology..

[14] Squadrito G, Pollicino T, Cacciola I, Caccamo G, Villari D, La Masa T (2006). Occult hepatitis B virus infection is associated with the development of hepatocellular carcinoma in chronic hepatitis C patients.. Cancer..

[15] Fukuda R, Ishimura N, Hamamoto S, Moritani M, Uchida Y, Ishihara S (2001). Co-infection by serologically-silent hepatitis B virus may contribute to poor interferon response in patients with chronic hepatitis C by down-regulation of type-I interferon receptor gene expression in the liver.. J Med Virol..

[16] De Maria N, Colantoni A, Friedlander L, Leandro G, Idilman R, Harig J (2000). The impact of previous HBV infection on the course of chronic hepatitis C.. Am J Gastroenterol..

[17] Fabris P, Brown D, Tositti G, Bozzola L, Giordani MT, Bevilacqua P (2004). Occult hepatitis B virus infection does not affect liver histology or response to therapy with interferon alpha and ribavirin in intravenous drug users with chronic hepatitis C.. J Clin Virol..

[18] Kao JH, Chen PJ, Lai MY, Chen DS (2002). Occult hepatitis B virus infection and clinical outcomes of patients with chronic hepatitis C.. J Clin Microbiol..

[19] Toyoda H, Kumada T, Kiriyama S, Tanikawa M, Hisanaga Y, Kanamori A (2011). High ability to predict the treatment outcome of peginterferon and ribavirin combination therapy based on the reduction in HCV RNA levels at 4 weeks after starting therapy and amino acid substitutions in the hepatitis C virus in patients infected with HCV genotype 1b.. J Gastroenterol..

[20] Ishak K, Baptista A, Bianchi L, Callea F, De Groote J, Gudat F (1995). Histological grading and staging of chronic hepatitis.. J Hepatol..

[21] Bahramali G, Sadeghizadeh M, Amini-Bavil-Olyaee S, Alavian SM, Behzad-Behbahani A, Adeli A (2008). Clinical, virologic and phylogenetic features of hepatitis B infection in Iranian patients.. World J Gastroenterol..

[22] Ghany MG, Strader DB, Thomas DL, Seeff LB (2009). Diagnosis, management, and treatment of hepatitis C: an update.. Hepatology..

[23] Sagnelli E, Imparato M, Coppola N, Pisapia R, Sagnelli C, Messina V (2008). Diagnosis and clinical impact of occult hepatitis B infection in patients with biopsy proven chronic hepatitis C: a multicenter study.. J Med Virol..

[24] Georgiadou SP, Zachou K, Rigopoulou E, Liaskos C, Mina P, Gerovasilis F (2004). Occult hepatitis B virus infection in Greek patients with chronic hepatitis C and in patients with diverse nonviral hepatic diseases.. J Viral Hepat..

[25] Hui CK, Lau E, Wu H, Monto A, Kim M, Luk JM (2006). Fibrosis progression in chronic hepatitis C patients with occult hepatitis B co-infection.. J Clin Virol..

[26] Uchida T, Kaneita Y, Gotoh K, Kanagawa H, Kouyama H, Kawanishi T (1997). Hepatitis C virus is frequently coinfected with serum marker-negative hepatitis B virus: probable replication promotion of the former by the latter as demonstrated by in vitro cotransfection.. J Med Virol..

[27] Branco F, Mattos AA, Coral GP, Vanderborght B, Santos DE, Franca P (2007). Occult hepatitis B virus infection in patients with chronic liver disease due to hepatitis C virus and hepatocellular carcinoma in Brazil.. Arq Gastroenterol..

[28] Caviglia GP, Abate ML, Manzini P, Danielle F, Ciancio A, Rosso C (2012). Occult hepatitis B virus infection in patients with chronic hepatitis C treated with antiviral therapy.. Hepat Mon..

[29] Selim HS, Abou-Donia HA, Taha HA, El Azab GI, Bakry AF (2011). Role of occult hepatitis B virus in chronic hepatitis C patients with flare of liver enzymes.. Eur J Intern Med..

[30] Kannangai R, Vivekanandan P, Netski D, Mehta S, Kirk GD, Thomas DL (2007). Liver enzyme flares and occult hepatitis B in persons with chronic hepatitis C infection.. J Clin Virol..

[31] Squadrito G, Orlando ME, Pollicino T, Raffa G, Restuccia T, Cacciola I (2002). Virological profiles in patients with chronic hepatitis C and overt or occult HBV infection.. Am J Gastroenterol..

[32] Sagnelli E, Coppola N, Scolastico C, Filippini P, Santantonio T, Stroffolini T (2000). Virologic and clinical expressions of reciprocal inhibitory effect of hepatitis B, C, and delta viruses in patients with chronic hepatitis.. Hepatology..

[33] Schmeltzer P, Sherman KE (2010). Occult hepatitis B: clinical implications and treatment decisions.. Dig Dis Sci..

[34] (2009). EASL Clinical Practice Guidelines: management of chronic hepatitis B.. J Hepatol..

[35] Kazemi-Shirazi L, Petermann D, Muller C (2000). Hepatitis B virus DNA in sera and liver tissue of HBsAg negative patients with chronic hepatitis C.. J Hepatol..

[36] Ke CZ, Chen Y, Gong ZJ, Meng ZJ, Liu L, Ren ZJ (2006). Dynamic changes of HBV DNA in serum and peripheral blood mononuclear cells of chronic hepatitis patients after lamivudine treatment.. World J Gastroenterol..

